# Role of the Chest Wall in Newborn Respiratory Function at Birth

**DOI:** 10.1096/fj.202502372R

**Published:** 2025-09-26

**Authors:** C. Diedericks, K. J. Crossley, I. M. Davies, D. A. Blank, S. J. E. Cramer, M. J. Wallace, A. B. te Pas, M. J. Kitchen, S. B. Hooper

**Affiliations:** ^1^ The Ritchie Centre Hudson Institute of Medical Research Clayton Victoria Australia; ^2^ Department of Obstetrics and Gynaecology Monash University Clayton Victoria Australia; ^3^ Monash Newborn, Monash Health Clayton Victoria Australia; ^4^ Division of Neonatology, Department of Paediatrics Leiden University Medical Centre Leiden the Netherlands; ^5^ School of Physics and Astronomy Monash University Clayton Victoria Australia

**Keywords:** chest wall compliance, chest wall mechanics, neonatal, respiratory transition

## Abstract

The chest wall significantly impacts respiratory function after birth, but its role in the newborn remains poorly understood as it is structurally and functionally different from adults. In neonates, the chest wall is highly compliant, which allows it to expand to accommodate the incoming air and the lung liquid cleared into the pulmonary interstitium during lung aeration. However, the high neonatal chest wall compliance predisposes it to distortion, which reduces breathing efficiency and necessitates respiratory muscle activation to stabilize it. This increases the work of breathing and, when combined with fewer fatigue‐resistant Type I muscle fibers (slow twitch, high oxidative capacity) in the diaphragm muscle, the risk of respiratory fatigue is increased. Nevertheless, as the chest wall is highly compliant in the newborn, recent studies have demonstrated that extra‐thoracic pressures can influence chest wall mechanics. Positive extra‐thoracic pressures (such as those applied with tight swaddling) limit chest wall expansion, whereas a small constant negative extra‐thoracic pressure stabilizes the chest wall and improves oxygenation in neonates. In this review, we aim to summarize the current evidence on chest wall function in fetuses and neonates, particularly during lung liquid clearance, lung aeration, and breathing after birth. Furthermore, we will explore how knowledge from newborn respiratory physiology may inform our understanding of the respiratory consequences of pulmonary oedema in adults, such as occurred during the initial stages of the COVID‐19 pandemic.

## Graphical abstract

At birth, liquid is cleared from the airways into lung tissue during lung aeration, causing the lungs to swell, which in turn causes the chest wall to expand. As the newborn chest wall is highly compliant, it can easily expand, but its high compliance reduces breathing efficiency after birth. In this review, we summarize the importance of the chest wall in the fetus and newborn, particularly during lung liquid clearance, lung aeration, and breathing after birth.

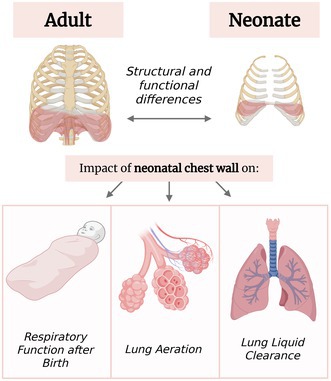

**Abbreviations**
BPD: bronchopulmonary dysplasia
CPAP: Continuous positive airway pressure
C_CW_
: chest wall compliance
C_L_
: lung compliance
C_RS_
: respiratory system compliance
FBM: fetal breathing movement
FRC: functional residual capacity
GA: gestational age
IP: Intrapleural
O_2_
: oxygen
PEEP: positive end‐expiratory pressure
PIP: Peak Inflation Pressure
P_IT_
: lung interstitial tissue pressure
RD: respiratory distress
REM: rapid eye movement

## 1 Introduction

The chest wall, which is primarily composed of the ribcage and diaphragm, is an integral component of the respiratory system. It provides a sealed chamber that houses and protects the lungs, preventing them from collapsing at end‐expiration, and generates the pressure gradients that draw air into the lungs during inspiration. However, until recently, its role in the adaptation of the respiratory system to air‐breathing at birth has been largely overlooked, most probably because the chest wall is considerably more compliant than the lung in the newborn due to its cartilaginous composition. Compliance refers to the elastic resistance of a structure [1], and, as the chest wall is highly compliant (very elastic) in newborns, the rib cage can deform during vigorous breathing. This reduces breathing efficiency and, therefore, is often perceived as a hindrance to the infant's ability to adapt to air breathing. However, recent studies have shown that high chest wall compliance is a critical feature of the respiratory transition at birth [2, 3].

The high compliance of the chest wall allows it to easily expand at birth to accommodate both the incoming air that generates the newly formed functional residual capacity (FRC) and the liquid that has moved from the airways into the lung tissue. Then, in the weeks after birth, the chest wall rapidly stiffens to increase breathing efficiency. By one year, the chest wall has stiffened so that chest wall compliance and lung compliance are approximately equal [4, 5]. This changing characteristic of the chest wall and its role in the respiratory adaptation to air breathing at birth is not well known or understood, yet it may help interpret the respiratory problems some infants suffer after birth. This review aims to consolidate recent knowledge on the role of the chest wall and formulate a new understanding of its role during the transition to air breathing at birth.

## 2 Respiratory System Mechanics in Adults

**FIGURE 1:**
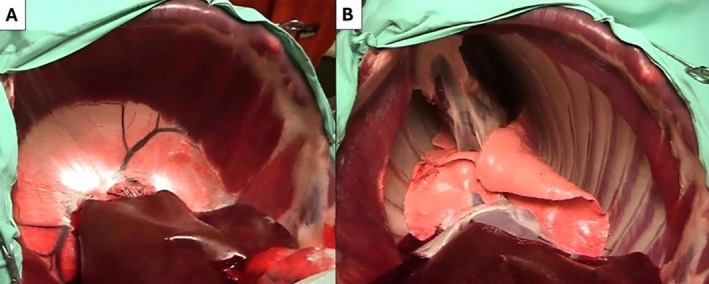
The impact of chest wall recoil on lung recoil. A and B are of a sheep chest and diaphragm viewed from the abdomen, below the liver, and oriented cranially. (A) The diaphragm is intact, the chest wall is “closed,” and the costal muscle of the diaphragm and the central tendon are clearly visible. A closed chest wall opposes the inward recoil of the lungs, causing the lungs to occupy almost the entire thoracic cavity. (B) The diaphragm has been cut, allowing air to enter the chest. An open chest wall causes the lungs to collapse as the inward recoil of the lungs is unopposed.

The respiratory system is comprised of the lungs and chest wall. The chest wall encapsulates the lungs and heart within a sealed chamber that can develop large increases and decreases in pressure (> 100 mmHg). In most mammalian species, the lungs and chest wall are separated by a liquid‐filled space called the intrapleural (IP) cavity, although in elephants, the two pleural membranes fuse in late fetal development, thereby losing this cavity [6]. The intrapleural cavity is lined by the visceral pleura, a fibrous membrane that lines the outer surface of the lungs, and the parietal pleura, which lines the inner surface of the chest wall. The pressure within the IP cavity is sub‐atmospheric due to the opposing recoil of the chest wall and lungs. Recoil refers to the intrinsic tendency to revert to a preferred position. At rest, surface tension at the air/liquid interface within the lung, combined with the elastic recoil properties of lung tissue, increases the tendency for the lungs to collapse. As the chest wall is sealed and the IP space is liquid‐filled (liquids are incompressible and in‐expandable), the tendency for the lungs to collapse is opposed by the chest wall. Therefore, the chest wall is largely responsible for maintaining expiratory lung volumes in adults. This is evidenced by the fact that the lungs markedly collapse if the chest cavity is opened to atmospheric pressures, commonly referred to as a pneumothorax (Figure 1). Nevertheless, in opposing lung recoil, the chest wall is drawn in by the lungs, as indicated by the diaphragm doming into the chest cavity, which generates an opposing recoil favoring the expansion of the chest wall (Figure 1). At rest, the tendency of the lungs to collapse is exactly opposed by the tendency of the chest wall to expand, which generates a sub‐atmospheric pressure within the IP cavity and, importantly, within lung tissue [7].

During quiet breathing, the diaphragm contracts, resulting in its caudal displacement into the abdomen. This increases the volume of the chest and further reduces pressures (more sub‐atmospheric) in the IP space and lung tissue. During accentuated breathing, contraction of the external intercostal muscles, which lift and expand the ribcage, also contributes to the expansion of the thoracic cavity. Expansion of the chest wall expands the lungs, reducing the pressure within the alveolar space. The reduction in the alveolar pressure creates a pressure gradient between the mouth and the distal airways, promoting airflow into the lungs if the upper airways, particularly the larynx, are open. Thus, the chest wall plays a critical role in breathing by inflating the lungs during inspiration [8].

As the lungs expand during inspiration, their recoil tendency at end‐inspiration is considerably greater than at end‐expiration (FRC). Therefore, when the inspiratory muscles of the chest wall relax at the beginning of expiration, lung recoil is unopposed, which causes the lungs to deflate, bringing the chest wall with it. Lung deflation increases alveolar pressure, which creates a pressure gradient between the alveolus and mouth opening that facilitates gas flow out of the lungs. While lung recoil decreases as the lungs deflate, chest wall recoil increases. Therefore, lung deflation will continue until the recoil of the chest wall counterbalances lung recoil, thus preventing further lung deflation. At end‐expiration, the opposing recoils of the lungs and chest wall are at equilibrium, and the volume of gas in the lungs at this point is known as the FRC, which is ~30%–35% of total lung volume in healthy individuals [9]. During accentuated breathing, the activation of expiratory muscles (internal intercostals and abdominal muscles) causes the chest wall to contract at an accelerated rate. This, combined with the lungs' recoil properties, greatly increases airflow out of the lungs and can cause the lungs to deflate well below FRC to a volume known as the residual volume.

Compliance is the inverse of recoil and refers to the change in volume per unit change in pressure, providing a measure of the elastic resistance of a structure. To optimize breathing efficiency, the chest wall must be stiff enough to counteract the lungs' inward recoil and resist the force of the diaphragm as it contracts during accentuated breathing. Conversely, the chest wall must be compliant enough to allow contraction and expansion during breathing, driving inspiration but not impeding expiration [5]. In healthy adults, this is achieved by having a relatively stiff ribcage that is retracted below its neutral recoil position due to lung recoil. As a result, during normal tidal breathing, the chest wall usually does not expand much beyond its neutral recoil position and so offers little to no impediment to lung expansion. Similarly, the lungs must be compliant enough to easily expand during inspiration but retain sufficient recoil to drive expiration [5].

## 3 Fetal Breathing Movements and the Chest Wall

During fetal life, gas exchange occurs across the placenta, as the future airways of the lungs are filled with liquid and do not participate in gas exchange. Nevertheless, the fetus performs fetal breathing movements (FBM) from ~10 weeks of gestation to term [10, 11], which play an important role in regulating airway liquid volumes before birth [12, 13]. During the final trimester, FBMs occupy 20–30 min of each hour and are closely correlated with rapid eye movement (REM) sleep state [11]. FBMs produce rhythmic contractions of the diaphragm, which can result in large reductions in tracheal and intra‐thoracic pressures, but they are unable to move large volumes of liquid into the lower airways [13]. This is because of the relatively high viscosity of lung liquid compared to air, which greatly increases the resistance of moving liquid (compared to air) through the airways [13]. As the chest wall is highly compliant (see below), the pressure gradients generated during FBMs cause the chest wall to deform with each inspiration, resulting in the movement of only small volumes of liquid through the airways [12].

Along with inducing contractions of the diaphragm, FBMs also influence the upper airways. During apnoeic periods, the laryngeal adductor muscles are tonically activated, which causes the glottis to close and facilitates the accumulation of lung liquid (secreted by the lung) within the future airways. The accumulated liquid expands the lungs, as evidenced by a higher pressure in the airways compared to the surrounding amniotic sac, which plays a critical role in lung growth and development [12, 13]. Indeed, reducing lung expansion by draining lung liquid, or by bypassing the glottis and connecting the trachea to the amniotic sac, causes lung growth to cease [14]. During periods of FBMs, the laryngeal adductor muscles dilate in phase with diaphragmatic contractions, allowing liquid to leave the lungs via the trachea due to higher pressure in the airways [15]. Although this loss of lung liquid during FBMs is counterintuitive, as contractions of the diaphragm would be expected to increase liquid influx, the loss of liquid is reduced by diaphragmatic contractions, which helps to maintain fetal lung liquid volumes during FBM episodes [13]. Indeed, when the diaphragmatic contraction component of FBMs is abolished (by spinal cord transection) while retaining glottic activity (innervated by nerves within the vagal trunk), fetal lung liquid volumes decrease by 27%–53% and lung growth is similarly reduced [16].

During FBMs, the primary respiratory muscle activated is the diaphragm, although at different times, other respiratory muscles can be activated as would normally occur postnatally. However, movement of the chest wall during inspiratory efforts in the fetus is quite different from adults and more closely resembles the expiratory phase in adults [12, 17]. In the fetus, the thorax moves caudally and inwards during inspiratory efforts rather than cranially and outwards [12, 17]. This is largely due to the high compliance of the fetal chest wall and its inability to oppose diaphragmatic contractions. In addition, as the relatively high viscosity of lung liquid increases the resistance to moving liquid through the airways, it is much more difficult to expand the lung with each inspiration. Nevertheless, FBMs result in the rhythmic contractions of the respiratory muscles, including the diaphragm, and play an essential role in activating and “exercising” respiratory muscles in utero [17, 18, 19].

## 4 Role of the Chest Wall in the Respiratory Adaptation at Birth

All infants are born with liquid in their airways, which must be cleared from the airways into the surrounding lung tissue after birth, allowing the primary organ of gas exchange to shift from the placenta to the lungs [20]. Thus, a smooth transition from fetal to neonatal life is largely dependent on the rapid and successful clearance of liquid from the airways so that pulmonary gas exchange can commence [21]. Airway liquid clearance is achieved by three mechanisms, which have been discussed in detail elsewhere, and so will only be briefly discussed here [22]. These mechanisms are: fetal postural changes, airway epithelial sodium channel activation, and inspiratory efforts [23].

### 4.1 Lung Liquid Clearance at Birth

#### 4.1.1 Fetal Postural Changes

During labor and delivery, particularly following membrane rupture and amniotic fluid loss, uterine contractions increase dorso‐ventral spinal flexion of the fetus [24]. These postural changes increase intra‐abdominal pressure, which displaces the diaphragm into the thoracic cavity and increases intrathoracic pressure. As a result, lung liquid is expelled via the nose and mouth [25]. As the fetal respiratory system is highly compliant, only small changes in transpulmonary pressure are required to expel large volumes of lung liquid [25, 26, 27].

#### 4.1.2 Airway Epithelial Sodium Channels

During gestation, lung liquid is secreted across the pulmonary epithelium into the lung lumen due to an osmotic pressure gradient generated by Cl^−^ secretion [28]. However, adrenaline release during labor is thought to reverse this osmotic gradient due activation of sodium channels. This increases Na^+^ uptake from the lung lumen, which is actively transported into the lung interstitium [23, 27]. The net movement of Na^+^ into the interstitium reverses the osmotic gradient, favoring water movement from the airways into the pulmonary interstitium [23, 29]. Sodium channels account for < 5% of airway liquid clearance at birth [23].

#### 4.1.3 Inspiratory Efforts

The primary mechanism of liquid clearance after birth is the hydrostatic pressure gradients generated by inspiration [26, 30, 31]. The sub‐atmospheric pressures generated by inspiration drive the rapid movement of liquid distally through the airways and across the distal airway wall (~3 mL/kg/s), allowing the airways to aerate in < 3 min [26, 31]. Airway liquid clearance rates via this mechanism are ~1000 times greater than the maximum rate achieved by adrenaline‐induced sodium reabsorption (~10 mL/kg/h) [31].

### 4.2 Consequences of Airway Liquid Clearance After Birth

**FIGURE 2:**
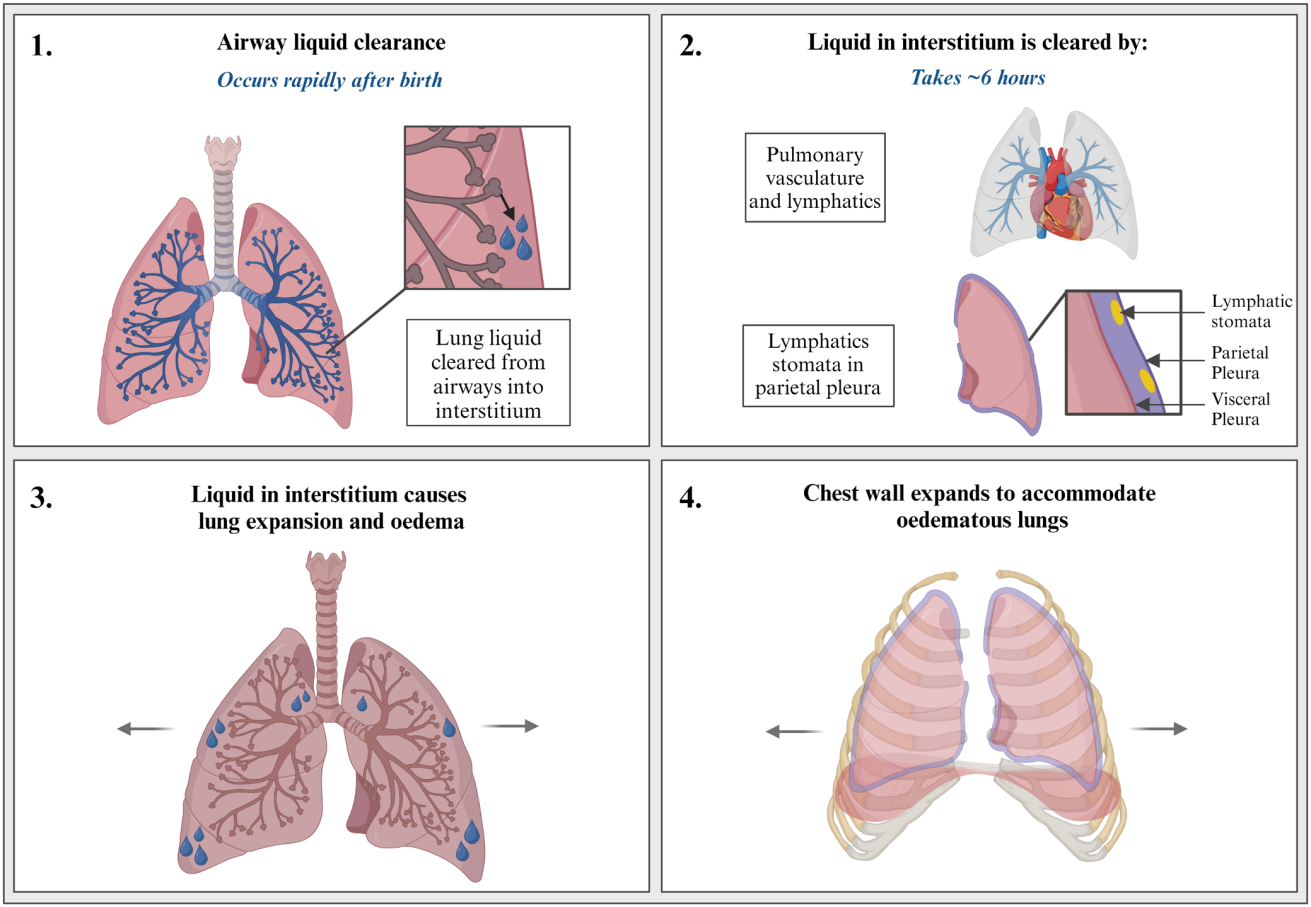
Schematic demonstrating the process of lung liquid clearance and the associated consequences. Once airway liquid is cleared into the pulmonary interstitium, the interstitium must be drained via pulmonary vasculature and lymphatics in a process that takes 4–6 h. As this process takes longer than the clearance of liquid from the airways, the lungs become oedematous and must expand. To accommodate this, the chest wall must also expand so that it can accommodate both this liquid and the incoming volume of air. Created with BioRender.com.

Irrespective of whether airway liquid is cleared after birth via inspiratory efforts or sodium reabsorption, the liquid cleared must enter the surrounding peri‐alveolar lung tissue, making this tissue oedematous (Figure 2). As all newborns are born with some liquid in their airways, and this liquid is cleared into lung tissue, all newborns have varying degrees of pulmonary oedema after birth [29, 30]. However, as airway liquid cleared via the nose and mouth avoids the need to clear this liquid into lung tissue, the larger the volume of airway liquid cleared via postural changes, the less oedematous the lung tissue will be after birth [32].

To resolve pulmonary oedema after birth, the liquid residing in pulmonary tissue must be cleared, which is a process that is thought to occur over the first 4–6 h after birth (Figure 2) [33, 34]. This liquid is thought to drain directly via either pulmonary lymphatics into the main thoracic lymph duct or via the pulmonary vasculature [33, 34, 35]. While the IP space typically contains a small volume of liquid that provides lubrication for the two pleural membranes, allowing the lung to slide smoothly along the inner surface of the chest wall during breathing, liquid residing within lung interstitial tissue is not cleared into the intrapleural space. Indeed, the movement of liquid into and out of the IP space occurs across the parietal pleura through stomata connected directly to lymphatics. These pores are unique to the parietal pleura [36] and pulmonary ligament, which is formed by the double‐layering of the parietal and visceral pleura [37]. Nevertheless, no liquid can move across the visceral pleura [38], and so the liquid in lung tissue must exit the lung via the capillaries or lymphatics.

The process of clearing liquid from the pulmonary interstitium after birth takes ~4–6 h, with ~92% of this liquid cleared by six hours after birth [34]. Thus, liquid drainage from the pulmonary interstitial tissue via lymphatics and vasculature is much slower than lung liquid clearance from the airways, which can occur in less than three minutes [30, 31, 39]. As such, the peri‐alveolar tissue must accommodate this liquid for several hours after birth. While this likely impacts respiratory function, the degree of impact will vary depending on the volume of liquid in the lung tissue [29, 30]. The main questions this raises are: (i) what determines the capacity of the lungs to accommodate this liquid and (ii) what impact does this liquid have on respiratory function after birth? The capacity of the lungs to accommodate airway liquid at birth depends on both the volume of liquid in the airways and the volume of peri‐alveolar lung tissue available to accommodate this liquid. During the last third of pregnancy, peri‐alveolar tissue volumes decrease exponentially relative to airway volume (indicated by the tissue: airway space ratio) [40]. As a result, ~80% of the lungs' volume is occupied by air within the airways at term birth [40]. This is largely due to distal airway growth and expansion, in conjunction with the condensation of peri‐alveolar tissue, which together drive the marked reduction in gas diffusion barriers needed for efficient gas exchange after birth [40]. Nevertheless, this leads to a simultaneous increase in airway volume, which is liquid‐filled, and a decrease in peri‐alveolar tissue volume with increasing gestation. Thus, as gestation progresses and the lung matures, potentially, an increasing volume of liquid must be accommodated in a decreasing lung tissue volume following lung aeration. This likely explains why increased airway liquid volumes have a greater impact on respiratory function in mature compared to immature (preterm) newborns [41]. It also underpins the importance of reducing airway liquid volumes via the nose and mouth before breathing onset at birth, particularly in more mature infants [31, 42].

### 4.3 Consequences of Lung Liquid Clearance and Aeration on the Chest Wall

**FIGURE 3:**
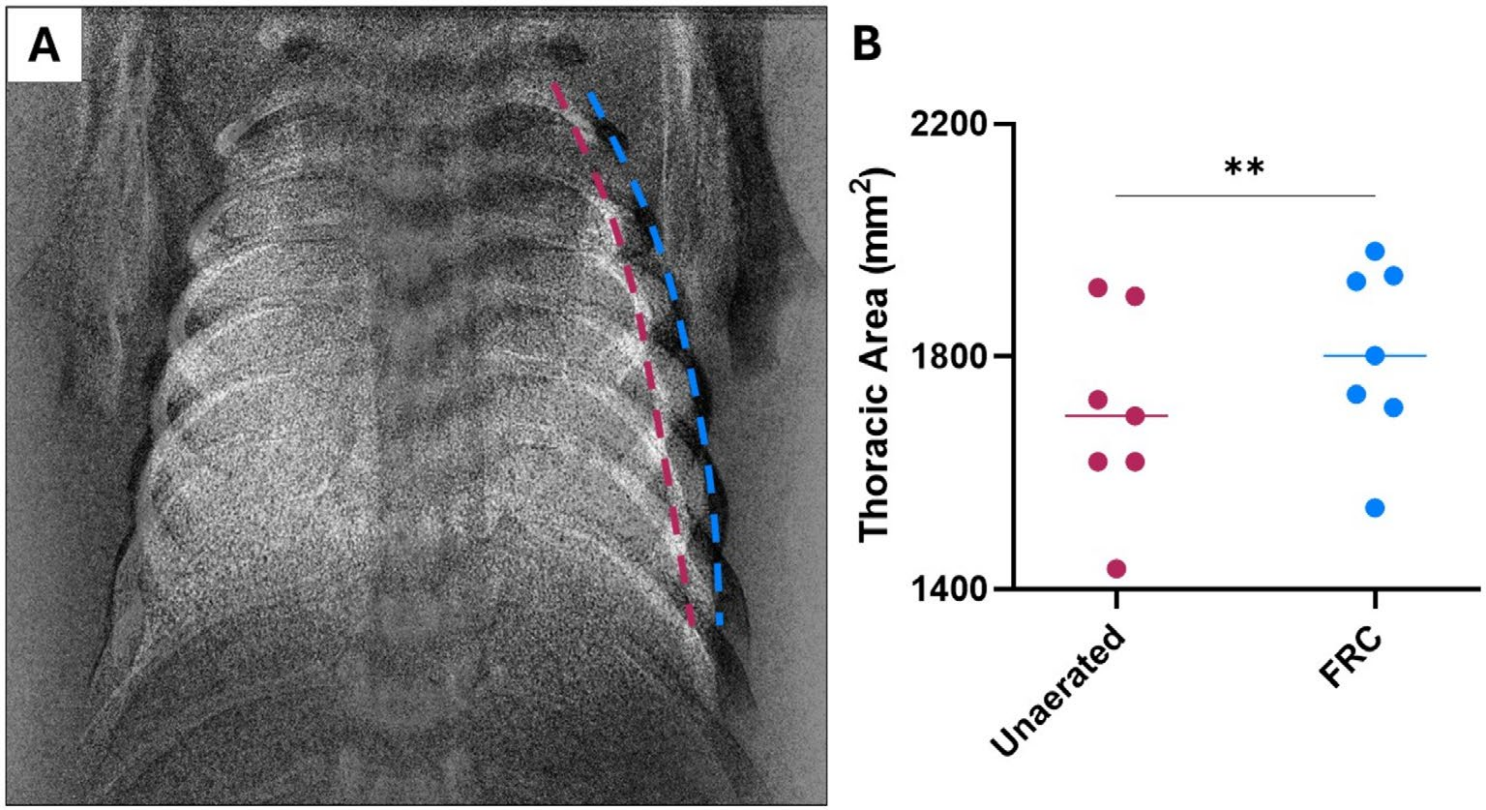
Thoracic expansion during lung aeration. (A) Overlaid images of thoracic area (mm^2^) before (magenta dashed line) and after (blue dashed line) lung aeration (functional residual capacity; FRC) are shown in newborn rabbit kittens. The kittens used for the overlain image had similar body weights. (B) shows the thoracic area before and after lung aeration. Data were analyzed with a paired t‐test and presented as individual data with the median. **p < 0.01. Data derived from and redrawn from Diedericks et al. [3].

To accommodate the movement of airway liquid into lung interstitial tissue at birth, the peri‐alveolar tissue compartment must expand, although the degree of expansion is likely to be small as this compartment is thought to be highly incompliant [42]. Nevertheless, as airway liquid has not left the thoracic area, the thoracic volumes must increase to also accommodate the incoming air that fills the airways after birth (Figure 2). The expansion of the chest wall after birth is indicated by Figure 3 [3, 30]. While this is commonly perceived to be a consequence of lung inflation, it is due to the combined effect of accommodating both the liquid that filled the airways and the incoming volume of air. As the neonatal chest wall is highly compliant at birth, expansion of the chest wall is easily achieved without substantially increasing intrathoracic pressures [32]. However, there must be a limit on how far the chest wall can expand before intrathoracic pressures substantially increase. Furthermore, while thoracic expansion at birth likely explains the evolutionary advantage of having a compliant chest wall, there is a “trade‐off.” The compliant chest wall reduces spontaneous breathing efficiency, which is commonly seen as chest wall retractions during respiratory distress [43].

## 5 The Role of the Chest Wall in Breathing in Neonates

### 5.1 Structure of the Chest Wall in Neonates

**FIGURE 4:**
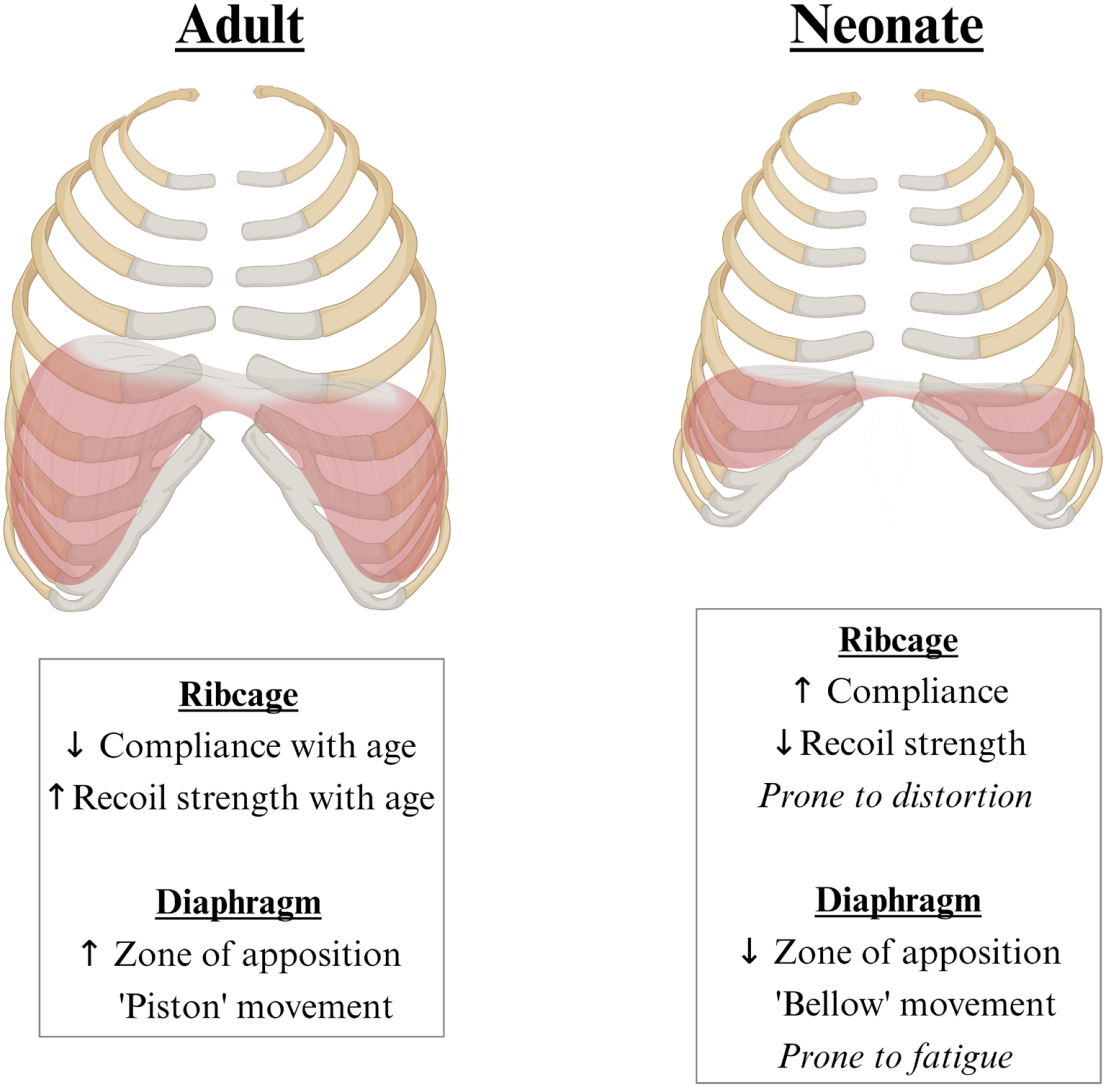
Chest wall and diaphragm anatomy in adults and neonates. Adults have an ellipsoid chest wall with a domed diaphragm, while neonates have a bell‐shaped chest wall with a flattened diaphragm. Created with BioRender.com.

In addition to different mechanics, the neonatal chest wall also exhibits distinct anatomical differences compared to adults (Figure 4). As the ribs primarily calcify after birth, the neonatal chest wall is comprised of ribs that are largely cartilaginous at birth. The cartilaginous composition of the ribs reduces their stability, making them more prone to ribcage distortion and increasing the need for respiratory muscle activation, both of which increase the work of breathing [5]. Furthermore, the chest wall is ellipsoid in adults and bell‐shaped in neonates, which reduces both anterior–posterior (pump‐handle) motion and transverse (bucket‐handle) expansion (at the costal margin) during inspiration, thus decreasing the thoracic expansion during inspiration [9]. As a result, neonates are more dependent on the diaphragm for breathing than adults [9]. However, the diaphragm is morphologically flatter in infants than adults, where the diaphragm domes up into the chest, resulting in a smaller zone of apposition in infants (Figure 4) [44]. In adults, the diaphragm moves like a piston during its contraction, with the apex descending towards the abdomen, whereas, in newborns, only the posterior region of the diaphragm moves caudally during contraction [45]. Thus, the movement of the diaphragm during breathing is more efficient in adults than in neonates. In addition, the neonatal diaphragm has fewer fatigue‐resistant Type I muscle fibers (~25% at term) than adults (~50%), which increases the risk of respiratory fatigue in the neonatal diaphragm [5, 18].

### 5.2 Compliance of the Respiratory System

In adults, while the chest wall and lungs have similar compliances, the chest wall is rigid enough to oppose the inward recoil of the lungs as well as the forces imposed on it by the contracting diaphragm during inspiration [5]. In neonates at birth, lung compliance (C_L_; mL/kg/cmH_2_O) is relatively low due to the presence of liquid in the airways/pulmonary interstitium and a reduced number of alveoli, whereas chest wall compliance (C_CW_; mL/kg/cmH_2_O) is high (relative to adults) due to the cartilaginous composition of the ribs [46, 47]. As a result, the chest wall is ~3 times more compliant than the lungs in term neonates [5, 46, 48]. However, to assist with breathing after birth, the C_CW_ must be low enough (i.e., rigid enough) to oppose the inward recoil of the lungs and contraction of the diaphragm [5]. Thus, while the high C_CW_ is essential for chest wall expansion during airway liquid clearance, it reduces the chest wall's ability to oppose lung recoil and counter the forces applied by the contracting diaphragm. As a result, resting lung air volumes are lower, and lung expansion during inspiration is less efficient in neonates than in adults [5].

### 5.3 The Impact of Neonatal Chest Wall Mechanics on Respiratory Function

**FIGURE 5:**
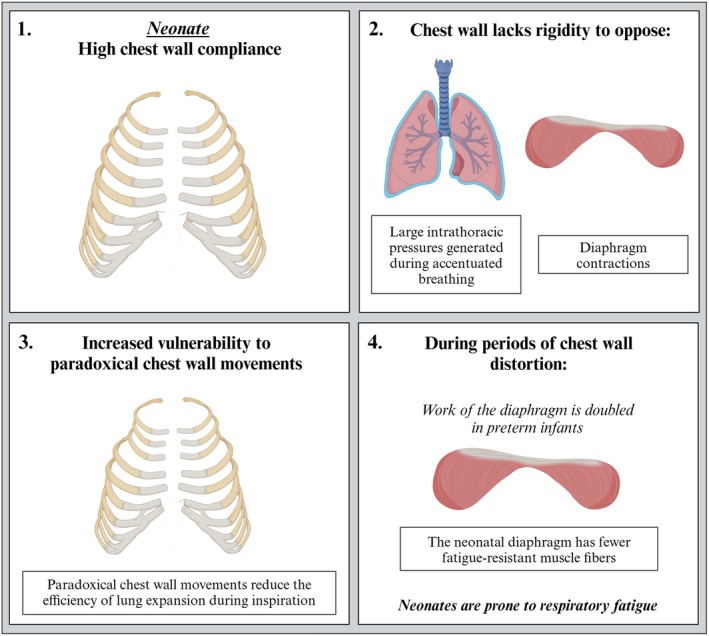
Destabilization of the chest wall in neonates. In neonates, the chest wall is highly compliant. The costal margins of the chest wall lack the rigidity to oppose the large intrathoracic pressure gradients generated during accentuated breathing by the diaphragmatic contractions. Thus, the costal chest wall margins move inwards with each inspiration. As the chest wall is more prone to distortion, energy expenditure during breathing is increased. However, neonates are more prone to respiratory fatigue due to fewer fatigue‐resistant muscle fibers in the diaphragm. Created in BioRender.com.

At birth, as the recoil strength (the inverse of compliance) of the lungs is considerably greater than that of the chest wall (Figure 5), the neutral recoil position of the lung and chest wall (i.e., the position at which chest wall recoil exactly counterbalances lung recoil) leads to a lower lung volume immediately after birth (10%–15% of total lung volume) than in day‐old newborns (20%–25%) and adults (30%–35% of total lung volume) [5, 9]. To counter this tendency for lower FRC levels, newborn infants often invoke active mechanisms to prevent regional atelectasis and to maintain lung volumes within a relatively compliant region of the pressure volume curve [5, 49]. These active mechanisms include expiratory braking maneuvers, which reduce/cease expiratory gas flow due to glottic adduction during middle to late expiration. Other mechanisms include high respiratory rates with shorter expiratory times and activation of intercostal muscles to stabilize the chest wall and prevent ribcage distortion [5, 9, 49, 50, 51].

### 5.4 Destabilization of the Chest Wall During Breathing

In neonates, a paradoxical *inward* recoil of the chest wall can occur when inspiratory efforts are accentuated, particularly if the infant has respiratory distress. As the costal margins of the chest wall lack the rigidity to oppose the contraction of the diaphragm, the chest wall margins move inward with each inspiration. Similarly, other chest wall regions (e.g., apical regions) also lack the rigidity to oppose the large intrathoracic pressures generated during accentuated inspiratory efforts and are drawn in. These paradoxical chest wall movements are relatively common in preterm infants as they have highly compliant chest walls and usually suffer from respiratory insufficiency due to lung immaturity, which reduces the lung's gas exchange capability [5, 46, 52, 53, 54]. When combined with the paradoxical motion of the chest wall during inspiration, the associated increased work of breathing increases the infant's energy requirement and need for oxygen when it is least able to provide it [5, 44, 46, 53, 54, 55, 56]. Indeed, during periods of ribcage distortion, the work of the diaphragm doubles in preterm infants (from 5.9 to 12.4 g cm mL^−1^) [44]. Furthermore, as the diaphragm of preterm infants has fewer fatigue‐resistant type 1 muscle fibers, the increased diaphragmatic workload increases the risk of respiratory fatigue in these infants [53, 55]. Thus, stiffening the chest wall after birth is a major requirement for improving breathing efficiency in these infants [44, 56].

### 5.5 Changes in Chest Wall Compliance After Birth

The energy expended during breathing rapidly decreases during the first two weeks after birth as ossification of the ribs stiffens the chest wall, resulting in a marked decrease in C_CW_ [4, 57]. Ribcage ossification continues at a slower pace for ~2 years until the compliances of the chest wall and lungs are approximately equal, as in adults [5]. While the factors regulating rib ossification are unknown, it is likely due to the forces imposed by lung recoil and breathing on the chest wall [4, 58]. Indeed, the effect of force application on calcium deposition in bone is well established [59]. Thus, it is possible that rib ossification may be delayed in infants exposed to mechanical ventilation strategies that oppose lung recoil for prolonged periods (e.g., PEEP and CPAP).

Long‐term mechanical ventilation also impacts diaphragmatic contraction strength in neonates, resulting in atrophied diaphragm musculature due to underuse [60]. While ventilator‐induced diaphragm dysfunction (VIDD) is also observed in adults, neonates are at greater risk of developing this problem [61]. Indeed, as infants are highly dependent on diaphragmatic activity for inspiration and to maintain FRC levels [5, 44], diaphragm atrophy increases the risk of extubation failure [61].

## 6 Altered Chest Wall Mechanics in Neonates

While respiratory mechanics are very different between adults and neonates, many states and conditions further influence how the respiratory system functions in neonates. This includes sleep, which impacts respiratory muscle activation and thus chest wall stabilization, or conditions that alter the compliance of the lungs or chest wall, such as prematurity, respiratory distress in term infants, congenital diaphragmatic hernia, and bronchopulmonary dysplasia.

### 6.1 Chest Wall Mechanics During Sleep

As respiratory muscle activation plays an important role in maintaining FRC levels in newborns, factors such as sleep states that reduce respiratory muscle activation can reduce FRC levels. For instance, during quiet sleep, the respiratory muscles are phasically activated [62, 63]. In contrast, during REM sleep, most respiratory muscle activity is suppressed, excluding the diaphragm, although its activity is also reduced, resulting in the potential for FRC loss in both adults and neonates [50, 63, 64]. However, the reduction in FRC levels is greater in neonates (~30%) than in adults (~10%) because of the instability of the neonatal chest wall [64, 65]. Furthermore, the absence of respiratory muscle activation to stabilize the chest wall during REM sleep increases the potential for chest wall distortion in infants [55, 66].

### 6.2 Prematurity

Chest wall compliance (C_CW_) decreases with increasing gestational age [51], decreasing from ~7.2 mL/(cmH_2_O*kg) in preterm infants (< 28 weeks gestation) to 4.2 mL/(cmH_2_O*kg) in term infants [51]. Conversely, C_L_ increases with increasing gestational age, due to distal airway growth and maturation, indicating that preterm infants have a C_L_ that is markedly lower than term infants [67]. As a result, C_CW_ is ~5 times greater than C_L_ in preterm infants but is only ~3 times greater in term infants [5, 51]. The combined effect of a high C_CW_ and a low C_L_ greatly reduces breathing efficiency in preterm infants as the chest wall is less able to oppose lung recoil and prevent distortion during inspiratory efforts. Further, when this reduced breathing efficiency is combined with an immature lung structure (specifically, a reduced gas exchange surface area and significantly increased air/blood gas diffusion distances) and an increased risk of respiratory fatigue [68], it is not surprising that most (92.6%) preterm infants require respiratory support after birth [69].

### 6.3 Bronchopulmonary Dysplasia

Infants born extremely preterm (< 28 weeks gestation) commonly develop bronchopulmonary dysplasia (BPD) in the weeks to months after birth. BPD is a chronic lung disease occurring primarily in preterm infants and is defined as the requirement for supplemental oxygen for > 28 days or at 36 weeks postmenstrual age. BPD is thought to arise due to lung injury, which is thought to alter the structural development of the lung, resulting in an ongoing reduction in respiratory function [70]. BPD also has significant impacts on both chest wall and lung mechanics, reducing C_L_, C_CW_, and FRC levels [48, 71, 72]. As BPD occurs postnatally and is not diagnosed until at least 28 days after birth (or 36 weeks postmenstrual age), the C_CW_/C_L_ ratio is the same as all preterm infants (5:1) immediately after birth. However, by 36 weeks postmenstrual age, C_CW_ is ~3 times greater than C_L_ (which is the expected C_CW_/C_L_ ratio in healthy term infants) [48].

In addition to reduced C_CW_, infants with BPD display asynchronous movement of the abdomen and chest wall during breathing, which is dependent on the degree of lung dysfunction [73]. Furthermore, chest wall abnormalities persist in infants with BPD for at least one year corrected postnatal age, with these children presenting with a flattened chest wall [72]. This altered shape is likely a response to the lower compliance of the lungs, resulting in an inward movement of the chest wall [72]. This is consistent with the concept that lung recoil plays an important role in determining chest wall stiffness and shape.

### 6.4 Respiratory Distress in Term Infants

Neonatal respiratory distress is most commonly associated with preterm infants but nevertheless accounts for ~51% of term infants admitted to intensive care [69]. However, the cause of respiratory distress is very different in term and preterm infants. In preterm infants, respiratory distress is caused by immature and surfactant‐deficient lungs, while in term infants, it is predominantly caused by the presence of too much liquid in the airways at birth [43, 74]. As all of this liquid must be cleared from the airways into the surrounding lung tissue, the degree of pulmonary oedema that develops after birth is markedly greater [74].

**FIGURE 6:**
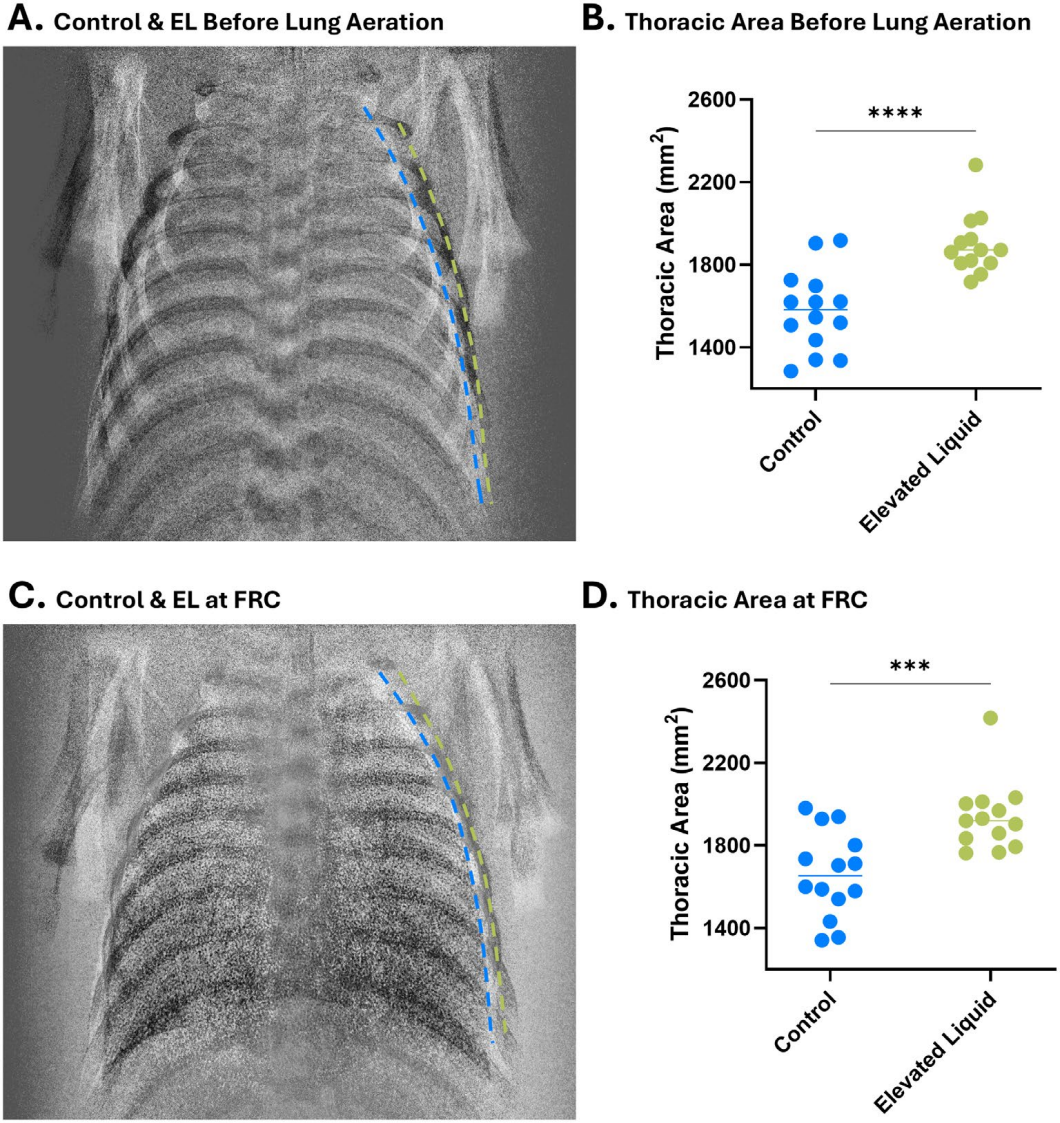
The effect of airway liquid volumes on thoracic area (mm^2^) standardized by the distance between the 1–5th vertebrae. Overlaid images of thoracic area are shown in newborn rabbit kittens with lung liquid volumes that would be expected following a vaginal delivery (~7 mL/kg; *Control*; blue dashed line) and volumes expected following caesarean section in the absence of labour (~37 mL/kg; *Elevated Liquid (EL)*; green dashed line) (A) before lung aeration and (C) after lung aeration, imaged at functional residual capacity (FRC). Kittens with matched body weights were used for the overlain images. The thoracic area (B) before lung aeration and (D) after lung aeration, measured at FRC are shown in *Control* kittens and kittens with elevated airway liquid volumes. FRC levels were 7.8 ± 3.3 mL/kg less in kittens with elevated airway liquid volumes than *Control* kittens (*p* = 0.03). Data were analyzed with an unpaired t‐test and presented as individual data with the median. ***p < 0.001, ****p < 0.0001. Includes data re‐analyzed and redrawn from Diedericks et al. [3].

As the chest wall must expand to accommodate both the airway liquid and incoming air, the presence of elevated airway liquid volumes means that the chest wall must expand to a greater degree to establish the same lung gas volume (FRC) as infants without elevated airway liquid volumes (Figure 6) [2]. As this greatly decreases respiratory system compliance (C_RS_; combined C_CW_ and C_L_), it is possible that the chest wall expands beyond its neutral recoil position. Thus, rather than counterbalancing each other, the recoil of the chest and lungs becomes additive. As a result, near‐term infants with elevated airway liquid at birth must defend their FRC against the combined recoil activity of the chest wall and lungs. These defenses include increased levels of active mechanisms such as expiratory braking maneuvers and diaphragmatic contractions at end‐inspiration [43].

## 7 Factors That Can Influence Chest Wall Mechanics

### 7.1 External Positive Pressure

**FIGURE 7:**
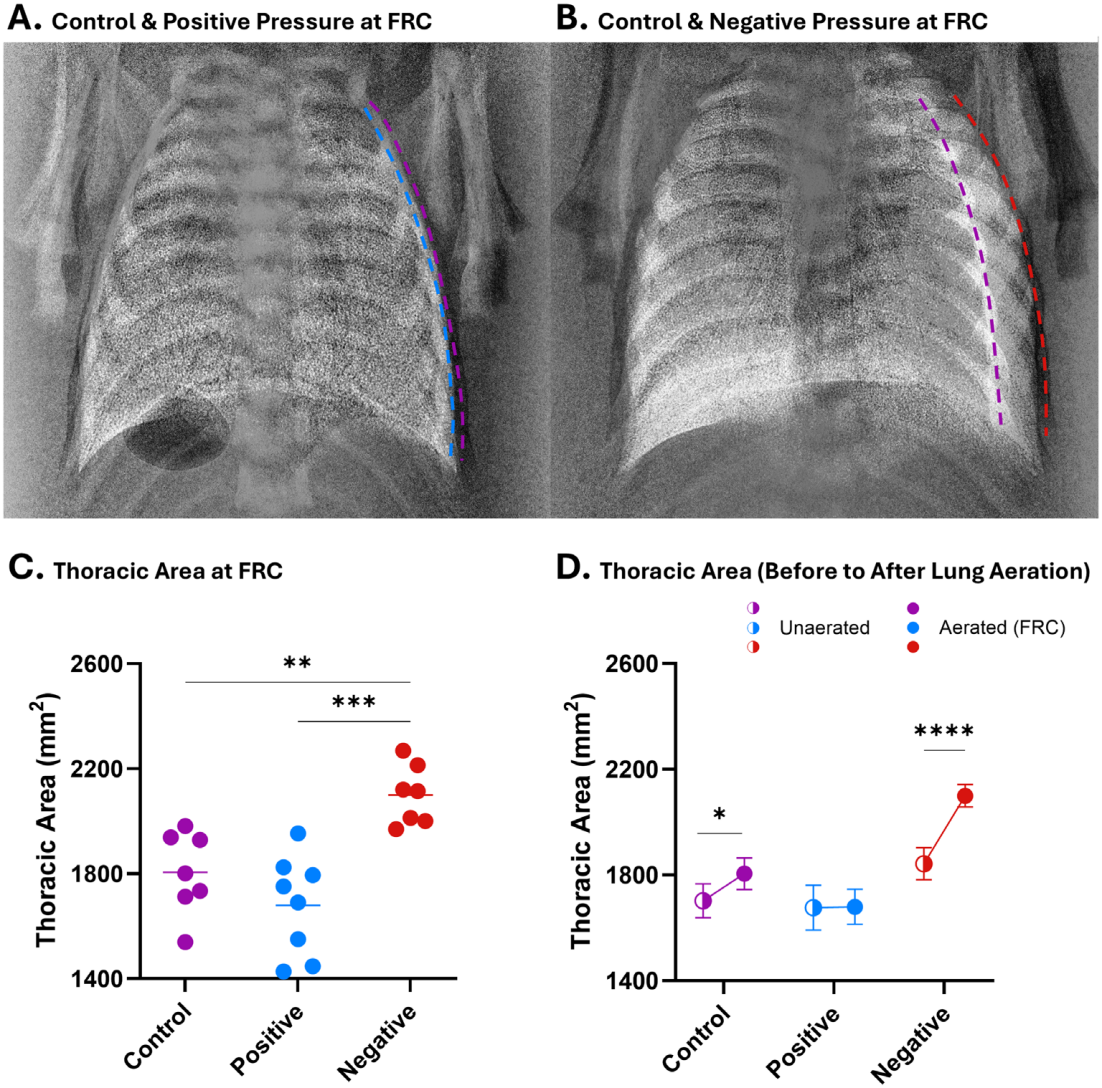
The impact that external positive (+6 cmH_2_O) and negative (−6 cmH_2_O) pressures have on thoracic area (mm^2^). Overlaid images of the thorax of newborn rabbit kittens acquired at maximum functional residual capacity (FRC) for kittens exposed to (A) external atmospheric (control; purple dashed line) and positive (blue dashed line) pressures and (B) external atmospheric (*Control*) and negative (red dashed line) pressures. Kittens with matched body weights were used for overlain images. (C) was analyzed with a one‐way ANOVA followed by Tukey's post hoc analysis and presented as the median, and (D) was analyzed with a two‐way ANOVA followed by Sidak multiple comparisons and presented as mean ± SEM. **p < 0.01; ***p < 0.001; ****p < 0.0001. Data are re‐analyzed and redrawn from Diedericks et al. [3].

Newborn infants are commonly and unintentionally exposed to procedures that can influence chest wall mechanics, including swaddling. Infants are commonly swaddled soon after birth to keep them warm and to provide comfort, but swaddling can apply an external positive pressure of 5–10 cmH_2_O to the chest wall, which may limit its capacity to expand (Figure 7) [75]. As chest wall expansion is an important component of the transition to air breathing at birth, swaddling may negatively impact the respiratory transition [2, 30]. Indeed, recent studies in newborn rabbits and sheep have demonstrated that an external positive pressure of ~6 cmH_2_O reduces C_RS_, increases peak inflation pressures (PIP) required to achieve a set tidal volume, reduces alveolar gas exchange, and reduces FRC levels immediately after birth [2, 3]. All of these changes were attributed to limiting the chest wall's ability to expand during, and shortly after, lung aeration (Figure 7D) [2, 3, 75]. These findings indicate that care should be taken not to swaddle infants too tightly in the immediate newborn period [2, 3].

### 7.2 External Negative Pressure

Negative pressure ventilation is not new and was commonly used to ventilate patients (in an “iron lung”) during the poliomyelitis epidemic from the 1930s to the 1950s [76]. It has also been used to ventilate infants with neonatal respiratory distress, but was superseded by continuous positive airway pressure [77]. Negative pressure ventilation transiently reduces the extra‐thoracic pressures (i.e., becomes increasingly negative) while the head and mouth remain at atmospheric pressure, thereby transiently reducing intrathoracic pressures and generating a pressure gradient for air to enter the lungs. In contrast to positive pressure ventilation, negative pressure ventilation more closely simulates normal breathing by creating sub‐atmospheric intrathoracic pressures to drive airflow into the lung. However, rather than trying to mimic breathing and ventilate the lungs, recent studies have examined whether small (approximately −6 cmH_2_O) continuous external negative pressures can expand and/or stabilize the chest wall in newborns receiving positive pressure ventilation (PPV) immediately after birth [2, 3]. Small continuous external negative pressures improved oxygenation and reduced C_CW_, stabilizing the chest wall and abolishing the need for an end‐expiratory pressure [2, 3]. It also expanded the chest wall (Figure 7B–D), reduced the PIP required to achieve a set tidal volume, increased FRC levels, and redistributed tidal ventilation into the larger, lower lung lobes [2, 3].

## 8 Pulmonary Oedema in Adults: Can We Learn From the Newborn?

While respiratory mechanics in adults and neonates are very different, the knowledge we have gained from how newborns transition at birth may help improve our understanding of the pathophysiology underpinning pulmonary oedema in adults. For instance, during the recent COVID‐19 pandemic, infection of the distal airways with the original strains of the SARS‐CoV‐2 virus caused severe pulmonary oedema and lung consolidation (airway flooding), particularly in dependent lung regions [78, 79, 80]. Thus, the initial respiratory pathology associated with COVID‐19 was physiologically similar to the respiratory distress suffered by term infants with elevated liquid in their airways at birth.

The initial COVID‐19 strains caused severe inflammation of the lungs, which increased vascular permeability in the lower airways [81]. This resulted in severe pulmonary oedema that, in some cases, increased lung weights by more than 1.5 kg, equating to ~15 mL/kg of liquid [82]. This is similar to (or less than) the expected volumes of liquid in the lungs after birth, which range from 7 to 14 mL/kg (infants born with labour) to ~37 mL/kg (infants born without labour; elective caesarean section) [74, 83]. As this liquid is captured in the lung, causing pulmonary oedema, the chest wall must expand to accommodate the increasing amount of liquid in lung tissue to maintain the same FRC levels. However, compared to the newborn, the adult chest wall is significantly stiffer (reduced C_CW_), with its stiffness increasing with age [84]. Thus, the ability of the chest wall to expand and accommodate additional volumes of liquid in lung tissue must be lower in adults than in newborns, leading to much higher tissue pressures for similar liquid volumes. Normally, lung interstitial tissue pressures (P_IT_) are sub‐atmospheric (up to −10 cmH_2_O in non‐dependent lung regions) [7], but pulmonary oedema, combined with the inability of the chest wall to expand easily, increases P_IT_ to supra‐atmospheric levels [42]. As this reverses the pressure gradient across the airway wall, this will facilitate liquid to leave the tissue and flood the airways, particularly in dependent lung regions [42, 82, 85]. Furthermore, as pulmonary lymphatic flow and drainage depend upon sub‐atmospheric P_IT_, supra‐atmospheric pressures would be expected to impair liquid drainage via the lymphatics, particularly as supra‐atmospheric lung tissue pressures will cause lymphatic vessels (and capillaries) to collapse. Indeed, this is consistent with the finding that pulmonary lymph vessels (and capillaries, as evidenced by clotting) were blocked in deceased COVID‐19 subjects [86]. As the liquid would become trapped in lung tissue following closure of the lymphatic vessels and capillaries, this would also explain why diuretics were largely ineffective at reducing the large pulmonary oedema suffered by COVID‐19 patients. Thus, it is interesting to speculate whether assisting chest wall expansion may have alleviated some of the respiratory distress suffered by COVID‐19 patients during the early phase of the epidemic.

## 9 Conclusions

This review has provided a comprehensive overview of the impact of the chest wall on the respiratory function of neonates. At birth, the chest wall must expand to accommodate both the liquid cleared from the airways into the pulmonary interstitium and the incoming air for sufficient pulmonary gas exchange. While a high chest wall compliance facilitates expansion, it also has adverse effects of reducing chest wall stability after birth, which impairs respiratory efficiency in newborns. In contrast, chest wall compliance is much lower in adults, which improves respiratory efficiency but reduces the capacity to cope with pulmonary oedema, such as is seen in patients with COVID‐19.

Despite its importance in neonatal respiratory function, the chest wall's impact continues to be overlooked. However, as modifying chest wall mechanics provides an alternative avenue for managing conditions such as respiratory distress in term infants, prematurity, and pulmonary oedema in adults, it should attract greater consideration in future research.

## Author Contributions

C.D., K.J.C., A.B.t.P., M.J.K. and S.B.H. contributed to the conception and planning of this review. C.D. and S.B.H. prepared the original manuscript, which was reviewed, edited, and approved by all authors prior to submission.

## Conflicts of Interest

The authors declare no conflicts of interest.

## Data Availability

Figure 6 includes previously published data supplemented with additional data, which will be made available upon reasonable request. Data sharing is not applicable to the remainder of this article, as no new data were created.
